# Socioeconomic inequalities in non-communicable diseases and their risk factors: an overview of systematic reviews

**DOI:** 10.1186/s12889-015-2227-y

**Published:** 2015-09-18

**Authors:** Isolde Sommer, Ursula Griebler, Peter Mahlknecht, Kylie Thaler, Kathryn Bouskill, Gerald Gartlehner, Shanti Mendis

**Affiliations:** Department for Evidence-based Medicine and Clinical Epidemiology, Danube University Krems, Dr.-Karl-Dorrek-Straße 30, 3500 Krems, Austria; Department of Anthropology, Emory University, 201 Dowman Drive, Atlanta, Georgia 30322 USA; Rollins School of Public Health, Emory University, 1518 Clifton Road Northeast, Atlanta, GA 30322 USA; RTI International, Research Triangle Park, 3040 East Cornwallis Road, Durham, NC USA; Chronic Disease Prevention and Management, World Health Organization, 20 Avenue Appia, Geneva, 1211 Switzerland

## Abstract

**Background:**

Non-communicable diseases (NCDs) are the largest cause of premature death worldwide. Socioeconomic inequalities contribute to a disparity in the burden of NCDs among disadvantaged and advantaged populations in low (LIC), middle (MIC), and high income countries (HIC). We conducted an overview of systematic reviews to systematically and objectively assess the available evidence on socioeconomic inequalities in relation to morbidity and mortality of NCDs and their risk factors.

**Methods:**

We searched PubMed, The Cochrane Library, EMBASE, SCOPUS, Global Health, and Business Source Complete for relevant systematic reviews published between 2003 and December 2013. Two authors independently screened abstracts and full-text publications and determined the risk of bias of the included systematic reviews.

**Results:**

We screened 3302 abstracts, 173 full-text publications and ultimately included 22 systematic reviews. Most reviews had major methodological shortcomings; however, our synthesis showed that having low socioeconomic status (SES) and/or living in low and middle income countries (LMIC) increased the risk of developing cardiovascular diseases (CVD), lung and gastric cancer, type 2 diabetes, and chronic obstructive pulmonary disease (COPD). Furthermore, low SES increased the risk of mortality from lung cancer, COPD, and reduced breast cancer survival in HIC. Reviews included here indicated that lower SES is a risk factor for obesity in HIC, but this association varied by SES measure. Early case fatalities of stroke were lower and survival of retinoblastoma was higher in MIC compared to LIC.

**Conclusions:**

The current evidence supports an association between socioeconomic inequalities and NCDs and risk factors for NCDs. However, this evidence is incomplete and limited by the fairly low methodological quality of the systematic reviews, including shortcomings in the study selection and quality assessment process.

**Electronic supplementary material:**

The online version of this article (doi:10.1186/s12889-015-2227-y) contains supplementary material, which is available to authorized users.

## Background

In 2012, non-communicable diseases (NCDs) were responsible for 38 million deaths globally [1]. The main chronic diseases contributing to NCD deaths were cardiovascular diseases (CVD), cancers, diabetes, and respiratory diseases, including asthma and chronic obstructive pulmonary disease (COPD). NCDs have historically been considered a problem of high income countries (HIC); however, the proportion of morbidity caused by NCDs in low and middle income countries (LMIC) is increasing [2]. Indeed, NCD mortality in LMIC now exceeds that of communicable diseases – with the exception of the African Region [1]. Based on recent estimates, nearly three quarters of all NCD deaths (28 million) were recorded in LMIC, 82 % of which were premature [1].

Behavioural risk factors for NCDs are well established and operate in a similar manner in all regions of the world [3]. The World Health Organization (WHO) has prioritised action on four risk factors that are shared among the four major NCDs: tobacco use, physical inactivity, the harmful use of alcohol, and unhealthy diets [1]. Six million people are currently estimated to die annually from tobacco use, and more than half of the deaths occurring in LMIC [1]. Likewise, physical inactivity caused 2.6 million deaths in LMIC and 0.6 million deaths in HIC. Harmful use of alcohol and unhealthy diets as measured by low fruit and vegetable consumption were also leading risk factors for mortality in middle income countries (MIC) and HIC [4].

Many studies have documented that lower socioeconomic status is associated with poorer health. Whilst this association is found in almost every country, the magnitude of inequality can be more pronounced in some countries than others [5, 6]. This is likely due to inequalities in living conditions more broadly that are generated by political, economic, social, and cultural factors [5]. For example, data derived from the 2002 to 2004 World Health Survey across LMIC showed that persons with lower wealth or education levels had higher prevalence of angina pectoris, arthritis, asthma, depression and comorbidity, but lower prevalence of diabetes than persons with higher wealth or education levels. At the same time, wealth and education inequalities were greater in low income countries (LIC) than MIC [2].

The aim of this work was to produce an overview of evidence on socioeconomic inequalities in the 1) incidence and prevalence as well as 2) adverse outcomes of the four main NCDs, and 3) incidence and prevalence of their risk factors, both within HIC and LMIC, and among them. This evidence is particulary important in terms of determining and monitoring inequalities in the burden of these diseases, and their risk factors across social groups and income levels of countries. This report is the first to summarise evidence on this level. We aimed to clarify what is already known about inequalities in NCDs, assess the magnitude of the problem, and highlight gaps in the evidence in order to inform priority areas for future research.

## Methods

### Search strategy

We searched PubMed, The Cochrane Library, EMBASE, SCOPUS, Global Health, and Business Source Complete from 2003 until December 2013. We limited electronic searches to human populations, systematic reviews, and English, German or Italian language (based on the language capabilities of the review team). Additionally, we screened reference lists of related narrative reviews identified during the study selection process. The full search strategy is presented in Additional file 1.

### Inclusion and exclusion criteria

We included only systematic reviews with a clear focus on inequality [7]. The factors determining socioeconomic status that we considered were: education, occupation, income, insurance status, and other indirect SES measures such as housing condition or maternal marital status [8]. We did not make any restrictions in the type of SES indicator, such as individual, household, neighbourhood or population level. We included systematic reviews on the following NCDs: CVD, cancers, type 2 diabetes, and chronic respiratory diseases (asthma and COPD), all of which account for the most NCD-related deaths globally [1]. Eligible risk factors for NCDs included: tobacco use, alcohol consumption, and obesity. We excluded reviews on specific populations such as prisoners, pregnant women, or children with disabilities.

### Selection strategy

Two persons independently reviewed abstracts and full-text articles against our pre-specified eligibility criteria. They resolved any discrepancies through discussion or consultation with a third reviewer.

### Data extraction and quality assessment

We extracted important information from included systematic reviews such as study populations, socioeconomic indicator, outcomes, and results. A second reviewer checked all abstracted data for completeness and accuracy.

To assess risk of bias (RoB) of systematic reviews, we used a modified version of the AMSTAR (A Measurement Tool to Assess Systematic Reviews) [9] appraisal tool. The tool consists of a 14-point checklist for the presence of a clearly defined research question, comprehensive and systematic literature search in multiple databases, dual and independent review and data abstraction of included studies, dual and independent assessment of quality of included studies, a clear definition of eligibility criteria, and, if a meta-analysis had been performed, an assessment of publication bias and testing, and an exploration of heterogeneity. We rated the reviews as low, unclear, or high RoB. Two independent reviewers assessed the RoB for each systematic review. Disagreements were resolved by discussion or by consulting a third reviewer.

In general terms, a review categorised as low RoB implies confidence that results represent true treatment effects. A review with unclear RoB is susceptible to some RoB but probably not enough to invalidate its results. A review assessed as high RoB has significant flaws of various types (e.g., stemming from serious errors in design, conduct, or analysis) that may invalidate its results.

## Results

**Fig. 1:**
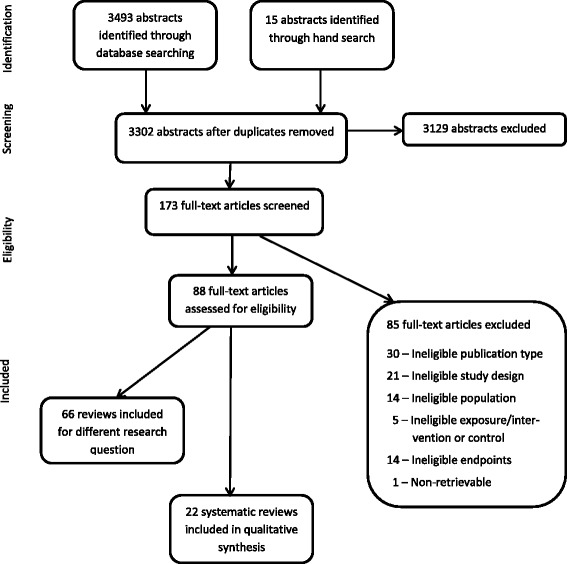
PRISMA Flow Diagram of the Study Selection Process

Out of 3302 abstracts, we identified 22 eligible systematic reviews for this research question. Figure 1 documents the study selection process of this report. Half of included studies [10–20], however, received a high RoB rating, mostly due to a lack of duality in the study selection process and an omission of a quality assessment of included studies. We narratively synthesized all included studies by outcome and order of NCDs or risk factors in Tables 1, 2 and 3 and below. The full data abstraction tables including RoB explanations can be found in Additional files 2, 3 and 4.

**Table 1:** Summary of results for incidence or prevalence of NCDs

Author, Year	Population	Outcome	Results	Risk of Bias
*Cardiovascular diseases*				
			Age-adjusted mean stroke incidence rates:	
Feigin et al., 2009 [24]	General population in HIC and LMIC	Incidence of stroke	*HIC:* 42 % reduction from 1970–79 to 2000–08 (*p* < 0.004); *LMIC:* >50 % increase from 1970–79 to 2000–08 (*p* < 0.0001)	Unclear
			Stroke mean incidence rates over time by age groups (1970–79 vs 2000–8):	
			*HIC:* 44 % (<75 y) and 41 % (> 75 y) reduction (both *p* < 0.0001); *LMIC:* 2-fold (< 75 y) and almost 4-fold (≥ 75 years) increase (*p* = 0.001 and *p* < 0.0001)	
Fowkes et al. 2013 [16]	General population in HIC and LMIC	Prevalence of PAD	Prevalence of PAD was higher in women from LMIC than HIC at all ages up to 60–64 years, above which the prevalence was higher in HIC. Prevalence of PAD was higher in men from HIC than LMIC at all ages.	High
			Rate of change from 2000 to 2010 was 28.7 % in LMIC and 13.1 % in HIC.	
Galobardes et al., 2006 [15]	General population mostly in HIC	Incidence or prevalence of overall CVD, CHD, stroke, other CVD subtypes	9 out of 9 prospective studies found a higher incident risk of CVD among those with low childhood SES.	High
			7 of 11 case-control studies showed an association of low childhood SES and risk for MI, angina, or stroke.	
			5 cross-sectional studies found a higher prevalence of CHD among those with low childhood SES.	
			HR for stroke incidence:	
Kerr et al., 2011 [23]	General population mostly in HIC (all but 1 study)	Incidence of stroke	*Unadjusted Meta-analysis (low vs high)* Education, occupation or income: 1.67, 95 % CI: 1.46–1.91	Unclear
			*Meta-analysis adjusted for grouped vascular risk factors (low vs high)* Education, occupation or income: 1.31, 95 % CI: 1.16–1.48	
			RR for acute MI:	
Manrique-Garcia et al., 2011 [22]	General population in HIC and LMIC	Incidence of acute MI	*Meta-analysis across countries (low vs high)* Education: 1.34, 95 % CI: 1.22–1.47; Occupation: 1.35. 95 % CI: 1.19–1.53; Income: 1.71, 95 % CI: 1.43—2.05	Unclear
			*HIC (low vs high)* Education: 1.39, 95 % CI: 1.25–1.55; Occupation: 1.41, 95 % CI: 1.25–1.59; Income: 1.76, 95 % CI: 1.46–2.12	
			*LMIC (low vs high)* Education: 1.16, 95 % CI: 0.97–1.39; Occupation: 0.51, 95 % CI: 0.27–0.99; Income:1.46, 95 % CI: 0.60–3.54	
Pollitt et al., 2005 [17]	General population from HIC	Incidence of CVD (MI, IHD, carotid IMT; CHD, AP), stroke	8 out of 9 studies found a higher incident risk of CVD among those with low childhood SES, but only few studies reported inverse adjusted (CVD risk factors and/or adult SES) associations. 2 out of 2 studies showed no significant associations between stroke risk and childhood SES. 1 study found an association between cumulative life course exposure to low SES conditions and increased CVD outcome.	High
Sposato et al., 2012 [21]	General population in HIC, MIC, and LIC	First-ever incidence of stroke	Lower PPP-aGDP correlated with higher incident risk of stroke (*ρ* = -0.661, *p* = 0.027).	Unclear
			Lower PPP-aTHE correlated with higher incident risk of stroke (*ρ* = -0.623, *p* = 0.040).	
			There were no correlations between unemployment rate and risk of stroke incidence (*ρ* = -0.492, *p* = 0.12).	
*Cancers*				
Adam et al., 2008 [11]	Children in HIC	Incidence of childhood leukaemia	Two studies showed an increased risk of leukaemia in children from deprived areas, 4 studies showed a decreased risk of leukaemia in children from deprived areas or lower SES, 1 study found SES not to be a determinant of leukaemia in children.	High
			RR for lung cancer incidence:	
Sidorchuk et al., 2009 [26]	General population in HIC and MIC	Incidence of lung cancer	*Meta-analysis, adjusted for smoking, RR (low vs high)* Education: 1.61, 95 % CI: 1.40–1.85; Occupation: 1.48, 95 % CI: 1.34–1.65; Income: 1.37, 95 % CI: 1.06–1.77	Unclear
			*HIC (low vs high)* Education: 1.66, 95 % CI: 1.10–2.51; Occupation: 1.42, 95 % CI: 1.26–1.62; Income: 1.39. 95 % CI: 1.13–1.69	
			*MIC (low vs high)* Education: 1.66, 95 % CI: 1.28–2.16; Occupation: 0.90, 95 % CI: 0.66–1.23; Income: 1.30, 95 % CI: 0.23–7.31	
Slatore et al., 2010 [25]	General population in the US	Incidence of lung cancer	1 study found higher incidence rates of lung cancer for women and men from all age groups with Medicaid insurance compared to non-Medicaid. 1 study found higher incidence rates of lung cancer for Medicare patients alone compared to Medicaid/Medicare patients, but the effect was removed when the comparison group was restricted to patients covered by Medicaid >12 months before diagnosis.	Unclear
			RR for lung cancer incidence:	
Uthman et al., 2013 [27]	General population in HIC, MIC, and LIC	Incidence of gastric cancer	*Overall (low vs high)* Education: 2.97, 95 % CI: 1.92–4.58; Occupation: 4.33, 95 % CI: 2.57–7.29; Income: 1.25, 95 % : 0.93–1.68; Combined SEP: 2.64, 95 % CI:1.05–6.63	Unclear
			*HIC (low vs high)* Education: 2.65, 95 % CI 1.64–4.30; Occupation: 6.79, 95 % CI 3.42–13.50; Income: 1.09, 95 % CI: 0.76–1.56; Combined SEP: 4.50, 95 % CI: 0.84–24.16	
			*MIC (low vs high)* Education: 5.11, 95 % CI 2.71–9.65; Occupation: 3.06, 95 % CI 2.10–4.8; Income: 1.48, 95 % CI: 0.61–3.58; Combined SEP: 1.36, 95 % CI: 0.52–3.60	
*Type 2 diabetes*				
			RR for type 2 diabetes incidence:	
Agardh et al., 2011 [28]	General population in HIC, MIC, and LIC	Incidence of type 2 diabetes	*Overall (low vs high)* Education: 1.41, 95 % CI: 1.28–1.51; Occupation: 1.31, 95 % CI: 1.09–1.57; Income: 1.40, 95 % CI: 1.04–1.88	Unclear
			*HIC (low vs high)* Education: 1.45, 95 % CI: 1.28–1.63; Occupation: 1.31, 95 % CI: 1.05–1.63; Income: 1.40, 95 % CI: 0.81–2.42	
			*MIC (low vs high)* Education: 1.59, 95 % CI: 1.28–1.97; Occupation: 1.27, 95 % CI: 0.96–1.68; Income: 1.39, 95 % CI: 1.06–1.82	
			*LIC (low vs high; n = 1);* Education: –; Occupation: –; Income: RR 1.27, 95 % CI: 0.99–1.62	
Tamayo et al., 2010 [18]	General population in HIC and MIC	Incidence of type 2 diabetes in later life	4 out of 6 studies showed an increased risk of type 2 diabetes in either girls or boys from low parental occupational status, 2 studies showed no association. For education, 2 out of 3 studies showed an increased risk of type 2 diabetes in children from low SES. 1 study showed no statistically significant association between type 2 diabetes incidence and childhood adversity.	High
*Chronic respiratory diseases*				
Gershon et al., 2012 [29]	General population in HIC	Prevalence and incidence of COPD	6 out of 8 studies found individuals of the lowest SES strata more likely to have or develop.	Low
			COPD than those of the highest (point estimates of OR ranging from 0.8–3.7, RII ranging from 2.2 to 3.2).	

*RR* Relative risk, *CI* Confidence interval, *SES* Socioeconomic status, *COPD* Chronic obstructive pulmonary disease, *OR* Odds ratio, *RII* Relative index of inequality, *na* not available, *SEP* Socioeconomic position, *y* years, *vs* versus, *p* p-value, *LMIC* Low and middle income countries, *LIC* Low income countries, *MIC* Middle income countries, *HIC* High income countries, *PAD* Peripheral artery disease, *MI* Myocardial infarction, *CVD* Cardiovascular diseases, *CHD* Coronary heart disease, *IHD* Ischaemic heart disease, *IMT* Intima-media thickness, *AP* Angina pectoris, *PPP-aGDP* Per capita GDP adjusted for purchasing power parity, *PPP-aTHE* total health expenditures per capita at purchasing power parity, *ρ* Spearman rank correlation coefficient, *n* number of studies, *NCDs* Non-communicable diseases, *HR* Hazard ratio

^a^Relative Index of Inequality is an indicator of the degree of inequality across socioeconomic categories

**Table 2:** Summary of results adverse outcomes from NCDs

Author, year	Population	Outcome	Results	Risk of Bias
*Cardiovascular diseases*				
			Risk of mortality in heart failure:	
Calvillo-King et al., 2012 [30]	Patients with heart failure in HIC	Mortality in heart failure after hospitalization (30 day)	Lower vs higher education: RR 1.05, 95 % CI: 0.98–1.12 (*n* = 1)	Unclear
			Lower vs higher neighbourhood SES: RR 1.13, 95 % CI: 0.92–1.38 (*n* = 1)	
			Medicaid insurance vs other: OR 0.66, 95 % CI: 0.3–1.4 (4 studies, result from one)	
			≤ 25 miles to hospital vs > 25 miles to hospital: OR 0.95, 95 % CI: 0.92–0.98 (*n* = 1)	
			Early case fatalities of total strokes (%):	
Feigin et al., 2009 [24]	Patients with stroke in HIC and LMIC	Early case fatality of stroke (21 day to 1 month)	*HIC:* non-significant reduction from 35.9 % (1970–79) to 19.8 % (2000–08)	Unclear
			*LMIC:* non-significant reduction from 35.2 % (1980–89) to 26.6 % (2000–08)	
Galobardes et al., 2006 [15]	General population mostly in HIC	Overall CVD, CHD, stroke, angina, other CVD subtypes mortality	19 out of 24 prospective studies found an association between low childhood SES and increased risk CVD mortality. In 5 out of 9 studies the association was stronger for stroke than CHD.	High
Galobardes et al., 2004 [10]	General population mostly in HIC	Overall CVD, CHD and stroke mortality	5 out of 9 studies found a higher risk of overall CVD mortality among those with low childhood SES, with results generally remaining statistically significant after adjustment for adult SES and/or adult CVD risk factors.	High
			7 out of 10 studies found a higher risk of CHD mortality among those with low childhood SES, although adult SES attenuated the association in some studies. 4 out of 6 studies found a higher risk of stroke mortality among those with low childhood SES.	
Pollitt et al., 2005 [17]	General population and patients with CVD and stroke from HIC	CVD, stroke mortality	11 out of 13 studies found a higher risk of CVD mortality among those with low childhood SES. Most associations remained statistically significant after adjustment for CVD risk factors and/or adult SES.	High
			3 out of 3 studies showed a higher risk of stroke mortality among those with low childhood SES. Adjustment for adjustment for CVD risk factors and/or adult SES had minor impact on the effect.	
			5 out of 5 studies reported an association between cumulative life course exposure to low SES conditions and increased CVD mortality.	
Sposato et al., 2012 [21]	Patients with stroke in HIC, MIC, and LIC	30-day case-fatality rates of stroke; intracerebral hemorrhages	Lower PPP-aGDP correlated with higher 30-day case-fatality rates of stroke (*ρ* = -0.713, *p* < 0.001) and a greater proportion of intracerebral hemorrhages (*ρ* = -0.689, *p* < 0.001).	Unclear
			Lower PPP-aTHE correlated with higher 30-day case-fatality rates of stroke (*ρ* = -0.701, *p* < 0.001) and a greater proportion of intracerebral hemorrhages (*ρ* = -0.643, *p* < 0.001).	
			There was no correlation between unemployment and 30-day case-fatality rates of stroke (*ρ* = 0.204; *p* = 0.32) and proportion of intracerebral hemorrhages (*ρ* = -0.258, *p* = 0.184).	
*Cancers*				
			Estimated survival of retinoblastoma:	
Canturk et al., 2010 [31]	Patients with retinoblastoma in upper MIC, lower MIC, and LIC	Survival of retinoblastoma	*Upper MIC:* 79 % (range, 54–93 %);	Unclear
			*Lower MIC:* 77 % (range, 60–92 %)	
			*LIC:* 40 % (range, 23–70 %) → *p* = 0.001	
Galobardes et al., 2004 [10]	General population mostly in HIC	Overall cancer, lung cancer, other cancers mortality	4 out of 5 studies found no association between overall cancer mortality and childhood SES, and the effect was removed by adjustment for adult SES in the remaining study. 3 out of 3 studies found a higher risk of lung cancer mortality among those with low childhood SES, although the association was largely explained by adults SES in 2 studies. 1 study showed no association of childhood SES with other smoking-related cancers.	High
			1 study found a higher risk of stomach cancer mortality among those with low childhood SES. 1 study found a higher risk of large-bowl and rectal cancer among those who had the poorest housing conditions during childhood.	
			There was no association between non-smoking related cancers (3 studies), prostate cancer (1 study) and malignant melanoma (1 study) mortality and childhood SES.	
Gorey et al., 2009 [20]	Patients with breast cancer in the US and Canada	Breast cancer survival	Within Canada, there was no association between area-SES and breast cancer survival, a little survival disadvantage was only observed for lowest vs. highest income areas (pooled RR 0.94, 95 % CI 0.93–0.95).	High
			Within the US, breast cancer survival was consistently associated with area-SES. Women with breast cancer from low and middle income areas had survival disadvantage compared to women from high income areas (pooled RR ranging from 0.73, 95 % CI 0.72–0.74 for low to 0.96, 95 % CI 0.94–0.98 for middle income area).	
Slatore et al., 2010 [25]	Patients with lung cancer in the US	Lung cancer mortality	4 out of 4 studies showed a higher risk for lung cancer mortality for Medicaid insurance vs. other or private insurance. 2 studies showed mixed results on the association between Medicare vs Medicaid/Medicare and lung cancer mortality. 1 study showed a higher risk for lung cancer mortality for Medicare insurance and no insurance compared to private insurance. 2 studies showed no association between lung cancer mortality and insurance status. 1 study found mixed results for lung cancer mortality and different Medicare schemes.	Unclear
*Chronic respiratory diseases*				
Galobardes et al., 2004 [10]	General population mostly in HIC	COPD mortality	1 study did not find an association between higher COPD mortality and overcrowding.	High
Gershon et al., 2012 [29]	Patients with COPD in HIC	COPD mortality	Individuals of the lowest SES consistently had significantly higher mortality from COPD than those of the highest, except for 1 study (out of 5) where income was not associated with COPD mortality.	Low

*SES* Socioeconomic status, *COPD* Chronic obstructive pulmonary disease, *RR* Relative risk, *OR* Odds ratio, *CI* Confidence interval, *p* p-value, *n* number of studies, *US* United States, *PPP-aGDP* Per capita GDP adjusted for purchasing power parity, *PPP-aTHE* total health expenditures per capita at purchasing power parity, *ρ* Spearman rank correlation coefficient, *y* years, *NCDs* Non-communicable diseases, *vs* versus, *LMIC* Low and middle income countries, *LIC* Low income countries, *MIC* Middle income countries, *HIC* High income countries, *PAD* Peripheral artery disease, *MI* Myocardial infarction, *CVD* Cardiovascular diseases, *CHD* Coronary heart disease, *IHD* Ischaemic heart disease, *IMT* Intima-media thickness, *AP* Angina pectoris

**Table 3:** Summary of results for incidence or prevalence of NCD risk factors

Author, year	Population	Outcome	Results	Risk of Bias
*Obesity*				
			Prevalence (overweight [obesity]) in urban area (*n* = 1):	
Ekpenyong and Akpan, 2013 [19]	Adults in Nigeria	Prevalence of overweight and obesity	Low SES: 24.8 % [12.9 %]; Medium SES: 18.9 % [5.7 %]; High SES: 14.6 % [4.9 %]	High
			Prevalence:	
Papandreou et al., 2008 [13]	Children and adults in Mediterranean countries	Prevalence of obesity	*Children* HIC vs MIC: 11.5 % vs 3.9 % (*p* = 0.071) [m]; 7.2 % vs 3.2 % (*p* = 0.074) [w]	High
			*Adults* HIC vs MIC: 20.1 % vs 22.0 % (*p* = 0.62) [m]; 24.4 % vs 30.2 % (*p* = 0.368) [w]	
Shrewsbury and Wardle, 2008 [12]	Children in HIC	Prevalence of childhood obesity	19 out of 45 studies found higher obesity prevalence rates among those with low SES (all indicators), 12 studies found no and 14 varied associations.	High
			15 out of 20 studies found higher obesity prevalence rates among those with low education, 1 study found no, and 4 varied associations.	
			5 out of 13 studies found higher obesity prevalence rates among those with low occupation, 6 studies found no, and 2 varied associations.	
			4 out of 11 studies found higher obesity prevalence rates among those with low income, 3 studies found no, and 4 varied associations.	
			2 out of 5 studies found higher obesity prevalence rates among those with low SES (composite measures), 1 study found no, and 2 varied associations.	
			2 out of 7 studies found higher obesity prevalence rates among those with low neighbourhood SES, 3 studies found no, and 2 varied associations.	
Tamayo et al., 2010 [18]	General population in HIC	Prevalence or incidence of overweight and obesity in later childhood/life	5 studies showed no direct or a small influence of education on later childhood overweight and obesity, 2 studies showed an increased risk for overweight and obesity in the lowest education strata.	High
			No or small associations between occupation and overweight or obesity were found in 4 studies, of which 2 reported on adult overweight and obesity. 2 studies reported an increased risk of later childhood overweight and obesity in the lowest occupation strata.	
			3 studies observed effects regarding income discrepancies and later childhood overweight and obesity, 2 studies showed no associations.	
			Prevalence ratios (NHANES^a^ data):	
Wang and Beydoun, 2007 [14]	US adults and children	Prevalence of overweight and obesity	*Adults (obesity)* Low vs high SES: 1.6 (m), 3.4 (w) in 1971–74; 1.1 (m), 1.3 (w) in 1999–2000.	High
			*Children aged 2–9 y (overweight)* Low vs high SES: 1.9 (m), 0.8 (f) in 1971–75; 1.8 (m), 1.0 (f) in -1999–2002.	
			*Children aged 10–17 y (overweight)* Low vs high SES: 0.8 (m), 2.0 (f) in 1971–75; 1.1 (m), 1.6 (f) in 1999–2002.	

*m* men or male, *w* women, *f* female, *SES* Socioeconomic status, *vs* versus, *US* United States, *y* years, *n* number of studies, *HIC* High income country, *MIC* Middle income country, *NCDs* non-communicable diseases

^a^National Health and Nutrition Examination Surveys (USA)

### Incidence or prevalence of non-communicable diseases

*Overall, the evidence suggests that having low SES and/or living in LMIC increases the risk of CVD, lung and gastric cancer, type 2 diabetes, and COPD.* We located a total of 14 systematic reviews [11, 15–18, 21–29] that investigated socioeconomic inequalities in relation to incidence or prevalence of NCDs. Seven of them looked for evidence on socioeconomic inequalities and CVDs [15–17, 21–24], four for inequalities in incidence or prevalence of different types of cancers [11, 25–27], another two for inequalities associated with diabetes risk [18, 28], and one for inequalities and chronic respiratory disease risk [29]. Although data from LMIC was generally scarce, seven systematic reviews provided between country comparisons based on income level [16, 21, 22, 24, 26–28] (Table 1).

#### Cardiovascular diseases

Whilst there are numerous systematic reviews on socioeconomic inequalities and CVD risk, all of them were fraught with some methodological shortcomings. We derived the best available evidence from three systematic reviews with an unclear RoB that focused on socioeconomic inequalities in incidence of stroke [23, 24] and acute myocardial infarction (MI) [22]. We located four additional systematic reviews with high RoB that reported on the prevalence or incidence of CVD and subtypes of CVD such as peripheral artery disease (PAD) and stroke [15–17, 21].

The evidence evaluating associations between SES and CVD risk is fairly consistent. Kerr et al. [23] pooled data from 12 studies conducted mostly in HIC and found a higher risk of stroke incidence in the lowest SES group as compared to the highest SES group (Hazard ratio [HR] 1.31). This was supported by Sposato and Saposnik [21] who reported a significant correlation between lower country-level macro-socioeconomic status indicators and higher risk of stroke incidence. Similarly, the review by Manrique-Garcia et al. [22] showed an overall increased risk for acute MI among the lowest SES group for income (Risk ratio [RR] 1.71), occupation (RR 1.34) and education (RR 1.35). When analysed by country's income level, associations between low SES and higher risk of acute MI incidence were significant in HIC but not LMIC. However, the vast majority of the 70 included studies stemmed from HIC. This imbalance in favour of studies from HIC was also observed by Feigin et al. [24], who analysed stroke incidence for four study periods and two country income levels. Over the four study periods, age-adjusted stroke incidence rates in HIC significantly decreased by 42 % (163 to 94 per 100000 person-years), while those in LMIC more than doubled and exceeded the rate in HIC (52 to 117 per 100000 person-years).

The three systematic reviews with high RoB largely support the findings from the three other systematic reviews or report similar results for other CVD subtypes [15–17]. However, the results from two systematic reviews on childhood socioeconomic inequalities and adult risk of CVD were mixed [15, 17]; the observed inverse associations were often attenuated or disappeared when adjusting for CVD risk factors or adult SES [17].

#### Cancers

We identified four systematic reviews on socioeconomic inequalities and cancer incidence, with two of them reporting on lung cancer [25, 26], one on gastric cancer [27], and another one on childhood leukaemia [11]. However, three of these reviews had an unclear RoB [25–27] and one a high RoB [11].

The systematic review of 64 studies by Sidorchuk et al. [26] constitutes the most comprehensive data collection on SES and lung cancer incidence to date. In their meta-analyses (adjusted for smoking), they found a statistically significant 1.37 (income) to 1.61 (education) higher RR for lung cancer in groups of low SES than those of high SES. The systematic review by Slatore et al. [25] of two studies on insurance status and risk of lung cancer in the US showed conflicting results.

The evidence base for SES and gastric cancer incidence is limited to an unclear-RoB systematic review encompassing 36 studies from MIC and HIC [27]. Its meta-analyses yielded results that are largely consistent with those on lung cancer; the pooled Relative Index of Inequality (RII) ranged from 2.97 (education) to 4.33 (occupation) for risk of developing gastric cancer in individuals from the lowest as compared to the highest SES group. Subgroup analysis at country income level showed mixed results, which might be explained by the uneven distribution of studies across country income level and SES indicators.

One systematic review (high RoB) on SES inequalities in relation to childhood leukaemia incidence found heterogeneous results and therefore no clear evidence to support an association [11].

#### Type 2 diabetes

For this outcome we located two systematic reviews. One review rated as unclear RoB and investigated associations between type 2 diabetes incidence and SES globally and subdivided by country income level [28]. Agardh et al. [28] pooled data from 23 studies and found an increased risk of type 2 diabetes for individuals in the lowest strata of education (RR 1.41), occupation (RR 1.31), and income (RR 1.40) as compared with individuals in the highest strata. Although the majority of studies were from HIC, subgroup analyses based on income level of countries supported the results in that the effect was consistent across LIC, MIC and HIC. The other systematic review was of high RoB and focused on childhood SES and risk for type 2 diabetes in HIC [18]. Six out of ten studies included in the systematic review by Tamayo et al. [18] indicate an association between childhood socioeconomic inequalities and risk of type 2 diabetes later in life.

#### Chronic respiratory diseases

Although we only identified one systematic review that met the eligibility criteria for chronic respiratory diseases, it is one of the few with low RoB in this overview. The majority of the studies (6 out of 8) included by Gershon et al. [29] found that individuals in the lowest SES group had a significantly increased risk of COPD when compared to those of the highest SES in HIC.

### Adverse outcomes from non-communicable diseases

*Systematic reviews show that low SES increases the risk of mortality from lung cancer, COPD, and reduces breast cancer survival in HIC. Early case fatalities of stroke are suggested to be lower and survival of retinoblastoma higher in MIC compared with LIC.*

Overall, we identified ten systematic reviews [10, 15, 17, 20, 21, 24, 25, 29–31] that examined associations between SES and adverse outcomes of NCDs. Among those ten, five focused on socioeconomic inequalities and mortality or fatality from CVDs [15, 17, 21, 24, 30], three on SES and adverse outcomes from cancer [20, 25, 31], and one on SES and chronic respiratory disease mortality [29]. One systematic review provided data on all those outcomes [10]. We could not find any evidence on socioeconomic inequalities and adverse outcomes from type 2 diabetes. Only two systematic reviews compared adverse NCD outcomes across country income levels (Table 2) [21, 24].

#### Cardiovascular diseases

The best available evidence of the six systematic reviews that met the eligibility criteria for CVDs can be derived from three unclear-RoB systematic reviews [21, 24, 30]. Results from these studies differed from the three remaining high-RoB systematic reviews [10, 15, 17].

In particular, Feigin et al. [24] found that early stroke case fatality rates (21 days to 1 month) from 2000 to 2008 were 25 % higher in LMIC than HIC (26.6 % vs 19.8 %). Likewise, lower country macro-socioeconomic status indicators but not unemployment rate was significantly correlated with higher 30-day case-fatality rates of stroke and a greater proportion of intracerebral haemorrhages in another systematic review [21]. The review by Calvillo-King et al. [30] showed no associations between short-term mortality in heart failure and education, neighbourhood SES, and insurance status (e.g. lower vs higher education: RR 1.05). The majority of studies included in the three other high-RoB reviews again found a greater risk for overall CVD, coronary heart disease or stroke mortality under poorer childhood socioeconomic circumstances, which was often attenuated by adult SES [10, 15, 17].

#### Cancers

The four systematic reviews that looked for evidence on SES and adverse cancer outcomes are heterogeneous in their study populations and outcomes, but rather homogenous in their results [10, 20, 25, 31]. We rated two of them as unclear RoB [25, 31] and two with high RoB [10, 20].

The review by Canturk et al. [31] found that survival of retinoblastoma was significantly lower in LIC as compared to MIC (40 % vs 77 %–79 %). Similarly, results of the review by Slatore et al. [25] were less conclusive but indicated high lung cancer mortality in patients on a social health care programme as compared to others. These findings were upheld by Galobardes et al. [10] who found that adult SES explained the observed associations between low childhood SES and increased risk of lung cancer mortality. No associations were seen between childhood SES and overall cancer mortality, as well as most other types of cancer mortality such as prostate cancer mortality. The review by Gorey et al. [20] showed that US women from low and middle income areas had worse 5-year breast cancer survival rates compared to high-income areas (pooled RR 0.73 and 0.96). In Canada, the survival disadvantage was minor and only observed in women from low compared to high-income areas (pooled RR 0.94).

#### Chronic respiratory diseases

Although we located two systematic reviews on SES in relation to chronic respiratory diseases, only one has a low RoB. This review reported significantly higher mortality from COPD for individuals of the lowest SES compared to the highest in HIC (4 out of 5 studies) [29]. The other review, however, has a high RoB and included data from only one study [10].

### Incidence or prevalence of risk factors for non-communicable diseases

Because we could not locate any systematic reviews on SES in relation to tobacco use or alcohol consumption, the available evidence is restricted to obesity (Table 3).

#### Obesity

*Systematic reviews included here indicate that lower SES is a risk factor for obesity in HIC, but this association varies by SES measure.*

We found five systematic reviews on SES in relation to prevalence or incidence of obesity, all of which were rated as high RoB [12–14, 18, 19]. Three of them assessed the impact of SES inequality on obesity in HIC [12, 14, 18], one in a LIC [19], and one compared obesity prevalence between high and middle income Mediterranean countries [13].

Tamayo et al. [18] and Shrewsbury and Wardle [12] drew from different study designs in order to investigate childhood SES inequalities in relation to childhood obesity. While both systematic reviews reported some studies that showed an increased risk or prevalence for childhood obesity in children with low parental occupation, the results from studies employing parental education or income as measures of SES are conflicting. Nevertheless, neither review located any studies demonstrating associations between high SES and increased risk or prevalence of childhood obesity.

In an earlier systematic review on US obesity data, Wang and Beydoun [14] reported higher prevalence rates of obesity among adults (ratio 1.1 in men and 1.3 in women), young boys (ratio 1.8), and adolescent girls (ratio 1.6) from the lowest SES group than individuals from the highest SES group. We located an additional systematic review that also compared obesity prevalence rates across age groups, but stratified by income levels of Mediterranean countries [13]. While obesity rates for children were lower in MIC than HIC (male 3.9 % vs 11.5 %; female 3.2 % vs 7.2 %), adults from MIC had higher obesity rates than those from HIC (male 22.0 % vs 20.1 %; female 30.2 % vs 24.4 %).

Only one systematic review collated evidence from LIC. Ekpenyong and Akpan [19] identified one eligible study from Nigeria showing that prevalence of overweight and obesity is higher in individuals in the lowest SES strata than in the highest SES strata (overweight [obesity]: 24.8 % [12.9 %] vs 14.6 % [4.9 %].

## Discussion

This overview of reviews aimed to provide a comprehensive overview of the evidence from systematic reviews on: the prevalence and incidence of NCDs, adverse outcomes from NCDs (mortality or survival time), and incidence or prevalence of risk factors for NCDs, by SES, and income level of countries. Overall, socioeconomic inequalities in relation to NCDs and their risk factors exist, but the located evidence is sparse and limited to only some NCDs in predominately HIC. A number of research gaps that require future investigation have arisen as a consequence thereof.

Systematic reviews on socioeconomic differentials and incidence or prevalence of NCDs suggest that low SES increases the risk for acute MI and stroke in HIC. Furthermore, evidence indicates that stroke incidence rates in LMIC are on the rise and exceed the rates in HIC, which are decreasing. In the case of adverse CVD outcomes, there is evidence suggesting that early case fatality rates of stroke are higher in LMIC than HIC, and there is insufficient evidence on socioeconomic inequalities and short-term mortality in heart failure in HIC. Although there is evidence to support increased risk of adult CVD incidence and mortality under poorer childhood socioeconomic circumstances, it was derived from high-RoB reviews and therefore constitutes an area for improvement. Similarly, other types of CVD incidences, prevalence rates or adverse outcomes in relation to SES and income level of countries have not been systematically investigated.

Systematic reviews that examined the association between cancer incidence or prevalence and socioeconomic inequalities are limited to lung and gastric cancers, demonstrating the need to examine other forms of cancer. These studies do however suggest that low SES is correlated with an increased risk for developing lung and gastric cancer. We did not anticipate locating a review on retinoblastoma, a rare childhood cancer caused by genetic mutation [32], suggesting that estimated survival is lower in LIC than MIC. In terms of other cancer outcomes, evidence is limited to lung cancer mortality and SES in HIC as all other associations on socioeconomic inequalities and cancer outcomes were reported in high-RoB reviews and do not provide sound evidence. Considering that liver, colorectal, and oesophageal cancers are also major causes of cancer-related mortality worldwide [33], more systematic reviews exploring the relationship between those cancers and socioeconomic inequalities or income levels of countries are warranted.

In regard to type 2 diabetes, available data from HIC and MIC indicates an increased risk for individuals from low SES as compared with individuals from high SES. Thus, data on diabetes risk and socioeconomic inequalities, as well as between socioeconomic inequalities and risk for type 2 diabetes later in life, present another gap in the evidence base. The only systematic review we identified on that topic was of high RoB and reported mixed results. Likewise, there is no systematic review on socioeconomic differentials and adverse outcomes from type 2 diabetes.

The strongest evidence of this overview comes from a low-RoB systematic review, which showed an increased risk for COPD incidence and mortality within low SES populations from HIC. In addition to the absence of evidence on social inequalities and COPD within LMIC, it is surprising that we did not identify any systematic review on asthma, which affects 300 000 million children and adults worldwide [34].

The topic most lacking in well-conducted reviews was socioeconomic disparities and risk factors for NCDs. Unexpectedly, we only found four systematic reviews on incidence or prevalence of obesity, all of which were of poor quality and included heterogeneous study designs, SES measures, populations, and age groups. As expected, the results from these reviews show a clear trend towards an association between low SES and increased risk of obesity. However, this trend needs to be confirmed by well-conducted systematic reviews on all age groups, including both childhood and adult SES. Further areas in need of review are the SES patterns of obesity in LMIC and comparisons of obesity prevalence and incidence between LMIC and HIC. We also found no reviews on SES inequalities in relation to tobacco use or alcohol consumption, both of which are important issues. In 2010, tobacco use (including second-hand smoke) (6.3 %) and alcohol use (5.5 %) followed hypertension (7.0 %) as the leading risk factors for the global disease burden [35].

Finally, it is worth noting that even though most reviews set out to include data from LMIC, they ended up with a strong imbalance in favour of studies from HIC. The lack of studies in LMIC points out the need for more research among these study populations in order to be able to assess socioeconomic inequalities within as well as among LMIC and HIC countries.

### Limitations

The major limitation of this overview is that the evidence is mainly based on poorly conducted systematic reviews. Out of 22, we identified a total of eleven systematic reviews [10–20] with shortcomings in the study selection and quality assessment process. Only one systematic review had a low RoB [29]. The limited number of well-conducted systematic reviews highlights the need for more reviews that apply rigorous methods. Another challenge common to all overviews of systematic reviews is publication bias. The search strategy was designed to include as many relevant systematic reviews as possible, but there might be others that we inadvertently omitted for this overview. Despite this inevitable fact, we believe that we have uncovered real gaps in the evidence base. Finally, we cannot rule out any potential overlap in the included primary studies between the systematic reviews that answered the same question regarding SES measures and outcomes. As this concerns mainly high-RoB systematic reviews whose results are deemed to be highly uncertain (for example Galobardes et al. [15] and Pollitt et al. [17]), the impact of the overlap on the evidence base is negligibly small.

## Conclusion

The systematic review evidence generally supports an association between socioeconomic inequalities and NCDs and/or risk factors for NCDs. However, this association is limited by the poor methodological quality of the reviews. Furthermore, there appear to be several gaps in the evidence base, which has led us to identify important topics for future research. This particularly concerns data from LMIC countries. Despite the efforts of authors of systematic reviews to include studies from LMIC, only a few were successful, thereby preventing any cross-country comparisons by country income level. There is also an unexpected lack of systematic reviews on socioeconomic inequalities and several types of cancers, asthma, and tobacco and alcohol use outcomes. Finally, it is important to compile evidence on childhood SES and NCDs and/or their risk factors, as the findings might be different than the associations with adulthood SES.

## Additional files

Additional file 1:
**Search Strategy.** (DOCX 21 kb) Additional file 2:
**Study characteristics and summary of results for incidence or prevalence of NCDs.** (DOCX 47 kb) Additional file 3:
**Study characteristics and summary of results for adverse outcomes from NCDs.** (DOCX 39 kb) Additional file 4:
**Study characteristics and summary of results for NCD risk factors.** (DOCX 27 kb)
