# Supplementary material for: Socioeconomic inequalities in non-communicable diseases and their risk factors: an overview of systematic reviews
Source: BMC Public Health. 2015 Sep 18;15:914. doi: 10.1186/s12889-015-2227-y (PMC4575459; doi:10.1186/s12889-015-2227-y)
Supplement: Additional file 1: — Search Strategy. (DOCX 21 kb) [file 12889_2015_2227_MOESM1_ESM.docx]

**Additional file 1: Search Strategy**

PubMed (Search conducted on December 21, 2013)

| [#1](http://www.ncbi.nlm.nih.gov/pubmed/advanced) |  | Search "Poverty"[Mesh] OR "Poverty Areas"[Mesh] OR (((("Income"[Mesh] OR "Socioeconomic Factors"[Mesh]) OR ( "Education"[Mesh] OR "Educational Status"[Mesh] )) OR "Occupations"[Mesh]) OR ( "Ethnology"[Mesh] OR "Ethnic Groups"[Mesh] )) OR ( "Residence Characteristics"[Mesh] OR "Emigrants and Immigrants"[Mesh] OR "Transients and Migrants"[Mesh] OR "Refugees"[Mesh] OR "Homeless Persons"[Mesh] ) | [996134](http://www.ncbi.nlm.nih.gov/pubmed/?cmd=HistorySearch&querykey=1) |
| --- | --- | --- | --- |
| [#2](http://www.ncbi.nlm.nih.gov/pubmed/advanced) |  | Search ((("Cardiovascular Diseases"[Mesh]) OR "Diabetes Mellitus"[Mesh]) OR "Neoplasms"[Mesh]) OR "Respiratory Tract Diseases"[Mesh] | [5051777](http://www.ncbi.nlm.nih.gov/pubmed/?cmd=HistorySearch&querykey=2) |
| [#3](http://www.ncbi.nlm.nih.gov/pubmed/advanced) |  | Search (("Smoking"[Mesh] OR "Smoking Cessation"[Mesh]) OR "Alcohol Drinking"[Mesh]) OR "Obesity"[Mesh] | [285659](http://www.ncbi.nlm.nih.gov/pubmed/?cmd=HistorySearch&querykey=3) |
| [#4](http://www.ncbi.nlm.nih.gov/pubmed/advanced) |  | Search ("Incidence"[Mesh]) OR "Prevalence"[Mesh] | [329608](http://www.ncbi.nlm.nih.gov/pubmed/?cmd=HistorySearch&querykey=4) |
| [#8](http://www.ncbi.nlm.nih.gov/pubmed/advanced) |  | Search (#1 AND #2 AND #4) Filters: Systematic Reviews; English; German; Italian | [206](http://www.ncbi.nlm.nih.gov/pubmed/?cmd=HistorySearch&querykey=8) |
| [#10](http://www.ncbi.nlm.nih.gov/pubmed/advanced) |  | Search (#1 AND #3 AND #4) Filters: Systematic Reviews; English; German; Italian | [93](http://www.ncbi.nlm.nih.gov/pubmed/?cmd=HistorySearch&querykey=10) |
| [#12](http://www.ncbi.nlm.nih.gov/pubmed/advanced) |  | Search (#1 AND #2) Filters: Systematic Reviews; English; German; Italian | [3215](http://www.ncbi.nlm.nih.gov/pubmed/?cmd=HistorySearch&querykey=12) |
| [#14](http://www.ncbi.nlm.nih.gov/pubmed/advanced) |  | Search ("Mortality"[Mesh] OR "mortality" [Subheading] OR "Mortality, Premature"[Mesh]) OR ( "Quality-Adjusted Life Years"[Mesh] OR "Quality of Life"[Mesh] OR "Life Expectancy"[Mesh] OR "Longevity"[Mesh] OR "Health Status"[Mesh] Filters: Systematic Reviews; English; German; Italian | [20624](http://www.ncbi.nlm.nih.gov/pubmed/?cmd=HistorySearch&querykey=14) |
| [#16](http://www.ncbi.nlm.nih.gov/pubmed/advanced) |  | Search (#12 AND #14) Filters: Systematic Reviews; English; German; Italian | [554](http://www.ncbi.nlm.nih.gov/pubmed/?cmd=HistorySearch&querykey=16) |
| [#18](http://www.ncbi.nlm.nih.gov/pubmed/advanced) |  | Search (#16 OR #10 OR #8) Filters: Systematic Reviews; English; German; Italian | [782](http://www.ncbi.nlm.nih.gov/pubmed/?cmd=HistorySearch&querykey=18) |
| [#20](http://www.ncbi.nlm.nih.gov/pubmed/advanced) |  | Search ("Health Expenditures"[Mesh] OR "Financing, Personal"[Mesh]) OR ( "Costs and Cost Analysis"[Mesh] OR "Hospital Costs"[Mesh] OR "Health Care Costs"[Mesh] ) OR "Health Services"[Mesh] OR "Delivery of Health Care"[Mesh] OR "Gross Domestic Product"[Mesh] OR "Cost Allocation"[Mesh] OR "Costs and Cost Analysis"[Mesh] OR "Cost of Illness"[Mesh] Filters: Systematic Reviews; English; German; Italian | [49071](http://www.ncbi.nlm.nih.gov/pubmed/?cmd=HistorySearch&querykey=20) |
| [#22](http://www.ncbi.nlm.nih.gov/pubmed/advanced) |  | Search (#12 AND #20) Filters: Systematic Reviews; English; German; Italian | [2306](http://www.ncbi.nlm.nih.gov/pubmed/?cmd=HistorySearch&querykey=22) |
| [#24](http://www.ncbi.nlm.nih.gov/pubmed/advanced) |  | Search (#18 OR #22) Filters: Systematic Reviews; English; German; Italian | [2644](http://www.ncbi.nlm.nih.gov/pubmed/?cmd=HistorySearch&querykey=24) |
| [#28](http://www.ncbi.nlm.nih.gov/pubmed/advanced) |  | Search (((("Early Medical Intervention"[Mesh]) OR "Community Health Planning"[Mesh]) OR "Public Health Surveillance"[Mesh]) OR "Health Policy"[Mesh] OR "Health Promotion"[Mesh] OR "Preventive Health Services"[Mesh] OR "Primary Prevention"[Mesh] OR "Secondary Prevention"[Mesh] OR "Tertiary Prevention"[Mesh] OR "Prevention and control [Subheading]"[Mesh]) Filters: Systematic Reviews; English; German; Italian | [12907](http://www.ncbi.nlm.nih.gov/pubmed/?cmd=HistorySearch&querykey=28) |
| [#30](http://www.ncbi.nlm.nih.gov/pubmed/advanced) |  | Search (#12 AND #28) Filters: Systematic Reviews; English; German; Italian | [1633](http://www.ncbi.nlm.nih.gov/pubmed/?cmd=HistorySearch&querykey=30) |
| [#32](http://www.ncbi.nlm.nih.gov/pubmed/advanced) |  | Search (#24 OR #30) Filters: Systematic Reviews; English; German; Italian | [2648](http://www.ncbi.nlm.nih.gov/pubmed/?cmd=HistorySearch&querykey=32) |
| #33 |  | Search (#3 AND #28) Filters: Systematic Reviews; English; German; Italian | 446 |
| #34 |  | Search (#24 OR #28 OR #30) Filters: Systematic Reviews; English; German; Italian | 2986 |
| #35 |  | Search (#24 OR #28 OR #30) Filters: Systematic Reviews; English; German; Italian; ("2004/01/01"[PDAT] : "2013/12/19"[PDAT]) | 2227 |

Cochrane (Search conducted on January 8, 2014)

(Noncommunicable or non-communicable or cardiovascular or cancer or "chronic respiratory" or "heart disease" or hypertension or "high blood pressure" or diabetes or asthma or COPD or "chronic obstructive pulmonary disease" or smoking or tobacco or obesity or overweight or alcohol or alcoholism):ti,ab,kw

AND

(poverty or education or income or occupation or inequality or ethnicity or gender or inequity or disparity or employment or "social class"):ti,ab,kw

AND

("health status" or mortality or incidence or prevalence or economics or "health care costs" or "health care cost" or "health care expenditures" or "health care expenditure" or "health care access" or intervention or program or "public health" or "premature death" or "life expectancy" or DALY or "disability adjusted life years" or "health promotion" or prevention or "gross domestic product" or GDP or "out of pocket" or "health service use"):ti,ab,kw

(restricted to 2003-2014 in Cochrane reviews (Reviews only), Other reviews and economic evaluations

407

EMBASE (Search conducted on December 21, 2013)

poverty or education or income or occupation or inequality or ethnicity or gender or geography OR inequity OR disparity OR employment OR “social class“ OR socioeconomic

AND

cardiovascular OR cancer OR respiratory OR non-communicable OR smoking OR obesity OR alcohol OR overweight

AND

"health status" or mortality or incidence or prevalence or economics or costs or expenditures or access or utilization or intervention or program or "public health"

AND Systematic Review/de AND **'human'**/de AND (**'controlled clinical trial'**/de OR **'evidence based medicine'**/de OR **'meta analysis'**/de OR **'randomized controlled trial'**/de) AND (**'asthma'**/de OR **'breast cancer'**/de OR **'cardiovascular disease'**/de OR **'diabetes mellitus'**/de OR **'heart failure'**/de OR **'heart infarction'**/de OR **'ischemic heart disease'**/de OR **'non insulin dependent diabetes mellitus'**/de OR **'obesity'**/de)

2003 forward = 352

Scopus (Search conducted on December 21, 2013)

  (**poverty** OR **education** OR **income** OR **occupation** OR **inequality** OR **ethnicity** OR **gender** OR **geography** OR **inequity** OR **disparity** OR **employment** OR**"social class"** OR **socioeconomic**) AND ((**noncommunicable** OR **non-communicable** OR **cardiovascular** OR **cancer** OR **"chronic respiratory"** OR **coronary** OR**cardiovascular** OR **“heart** **attack”** OR **“heart** **disease”** OR **“myocardial** **infarction”** OR **stroke** OR **cerebrovascular** OR **thrombosis** OR **hypertension**)) AND(**"health status"** OR **mortality** OR **incidence** OR **prevalence** OR **economics** OR **costs** OR **expenditures** OR **access** OR **utilization** OR **intervention** OR **program** OR**"public health"** OR **“premature** **death“** OR **“life** **expectancy“** OR **daly** OR **“disability** **adjusted** **life** **years“**) AND (LIMIT-TO(DOCTYPE, **"re"**)) AND (LIMIT-TO(LANGUAGE,**"English"**) OR LIMIT-TO(LANGUAGE, **"German"**) OR LIMIT-TO(LANGUAGE, **"Italian"**)) AND (LIMIT-TO(PUBYEAR, **2014** - **2003**)) = 364

Global Health (Search conducted on December 21, 2013)

poverty or education or income or occupation or inequality or ethnicity or gender or geography OR inequity OR disparity OR employment OR “social class“ OR socioeconomic

AND

cardiovascular OR cancer OR respiratory OR non-communicable OR smoking OR obesity OR alcohol OR overweight

AND

"health status" or mortality or incidence or prevalence or economics or costs or expenditures or access or utilization or intervention or program or "public health"

AND Systematic Review, 2003 forward = 141

Business Source Complete (Search conducted on December 21, 2013)

poverty or education or income or occupation or inequality or ethnicity or gender or geography OR inequity OR disparity OR employment OR “social class“ OR socioeconomic

AND

cardiovascular OR cancer OR respiratory OR non-communicable OR smoking OR obesity OR alcohol OR overweight

AND

"health status" or mortality or incidence or prevalence or economics or costs or expenditures or access or utilization or intervention or program or "public health"

AND Review, 2003 forward = 2
