# Supplementary material for: Socioeconomic inequalities in non-communicable diseases and their risk factors: an overview of systematic reviews
Source: BMC Public Health. 2015 Sep 18;15:914. doi: 10.1186/s12889-015-2227-y (PMC4575459; doi:10.1186/s12889-015-2227-y)
Supplement: Additional file 2: — Study characteristics and summary of results for incidence or prevalence of NCDs. (DOCX 47 kb) [file 12889_2015_2227_MOESM2_ESM.docx]

Additional file 2: Study characteristics and summary of results for incidence or prevalence of NCDs

| Author, Year | Population  SES indicator (level) | Outcome | Results | Risk of Bias | | |
| --- | --- | --- | --- | --- | --- | --- |
| *Cardiovascular diseases* | | | | |  |  |
| Feigin et al., 2009 [[24](#_ENREF_24)] | **Population**  General population in HIC and LMIC  **SES indicator (level)**  Country income [according to the World Bank’s country classification] (population) | Incidence of stroke (cases ascertained by multiple overlapping sources of information (hospitals, outpatient clinics) | Age-adjusted mean stroke incidence rates per 100000 person-years:  *HIC*  - 1970-79: 163, 95%CI: 98-277  - 2000-08: 94, 95%CI: 72-116  →42% reduction (p < 0.004)  *LMIC*  - 1970-79: 52, 95%CI: 33-71  - 2000-08: 117, 95%CI: 79-156  →more than double increase (p < 0.0001)  Analysis of stroke mean incidence rates over time by age groups (1970-79 vs 2000-8)  *HIC*  < 75 y: 118, 95%CI: 95-140 vs 66, 95%CI: 51-81  ≥ 75 y: 2044, 95%CI: 1754-2334 vs 1216, 95%CI: 998-1434  → 44% (< 75 y) and 41% (> 75 y) reduction (both p < 0.0001)  *LMIC*  < 75 y: 46, 95%CI: 26-66 vs 94, 95%CI: 70-118  ≥ 75 y: 340, 95%CI: 266-414 and 1030, 95%CI: 910-1151  →two fold (< 75 years; p = 0.001) and almost fourfold (≥ 75 years) increase (p < 0.0001) | Unclear (No quality assessment of included studies) | | |
| Fowkes et al. 2013 [[16](#_ENREF_16)] | **Population**  General population in HIC, MIC, and LIC  **SES indicator (level)**  Country income [according to the World Bank’s country classification] (population) | Prevalence of PAD (ankle brachial index, no measurement criteria) | Prevalence of PAD was higher in women from LMIC than HIC at all ages up to 60-64 years (LMIC 3.96-8.87% vs HIC 2.70-8.60%), above which the prevalence was higher in HIC (LMIC 10.08-24.20% vs HIC 9.91-18.65%). Prevalence of PAD was higher in men from HIC than LMIC at all ages (LMIC 1.21-21.50% vs HIC 2.76-24.77%) (no tests for statistical significance conducted).  Rate of change from 2000 – 2010 was 28.67% in LMIC and 13.08% in HIC. | High (No dual abstract review, no quality assessment of included studies) | | |
| Galobardes et al., 2006 [[15](#_ENREF_15)] | **Population**  General population mostly from HIC  **SES indicator (level)**  Education, occupation, wealth, employment, social class (maternal, paternal, parental), housing conditions, overcrowding, number of siblings, farm size, car ownership(household); maternal marital status, single-parent family and other indirect SES measures | Incidence or prevalence of CVD, CHD (ischaemic heart disease or myocardial infarction), stroke, angina, other CVD subtypes ( carotid intima-media thickness) | 9 out of 9 prospective studies found a higher incident risk of CVD among those with low childhood SES.  7 of 11 case-control studies showed an association of low childhood SES and risk for MI, angina, or stroke.  5 cross-sectional studies found a higher prevalence of CHD among those with low childhood SES. | High (Search databases not mentioned, no information on review process, no quality assessment of included studies) | | |
| Kerr et al., 2011 [[23](#_ENREF_23)] | **Population**  General population mostly in HIC (all but 1 study)  **SES indicator (level)**  Education, occupation or income (individual) | Incidence of stroke (self-reported, hospital discharge, medical records, death registry) | Risk for stroke incidence:  *Unadjusted Meta-analysis (low vs high)*  Education, occupation or income: HR 1.67, 95%CI: 1.46-1.91  *Meta-analysis adjusted for grouped vascular risk factors (low vs high)*  Education, occupation or income: HR 1.31, 95%CI: 1.16-1.48 | Unclear (No dual abstract review, no quality assessment of included studies) | | |
| Manrique-Garcia et al., 2011 [[22](#_ENREF_22)] | **Population**  General population in HIC and LMIC  **SES indicator (level)**  Education, occupation or income (individual or household) | Incidence of acute MI (assessment method not reported) | Risk for acute MI:  *Meta-analysis across countries (low vs high)*  Education: RR 1.34, 95%CI: 1.22-1.47  Occupation: RR 1.35. 95%CI: 1.19-1.53  Income: RR 1.71, 95%CI: 1.43—2.05  *HIC (low vs high)*  Education: RR 1.39, 95%CI: 1.25-1.55  Occupation: RR 1.41, 95%CI: 1.25-1.59  Income: RR 1.76, 95%CI: 1.46-2.12  *LMIC (low vs high)*  Education: RR 1.16, 95%CI: 0.97-1.39  Occupation: RR 0.51, 95%CI: 0.27-0.99  Income: RR 1.46, 95%CI: 0.60-3.54 | Unclear (Unclear if dual abstract review, no quality assessment of included studies) | | |
| Pollitt et al., 2005 [[17](#_ENREF_17)] | **Population**  General population in HIC  **SES indicator (level)**  Education, occupation, employment (parental/paternal); housing conditions, family size, perceived wealth, family situation, farm size (household) and other indirect SES measures | Incidence of CVD (MI, IHD, carotid IMT; CHD, AP), stroke  (self-report or assessment method not reported) | 8 out of 9 studies found a higher incident risk of CVD among those with low childhood SES, but only few studies reported inverse adjusted (CVD risk factors and/or adult SES) associations.  2 out of 2 studies showed no significant associations between stroke risk and childhood SES:  1 study found an association between cumulative life course exposure to low SES conditions and increased CVD outcome | High (Only one database searched) | | |
| Sposato et al., 2012 [[21](#_ENREF_21)] | **Population**  General population in HIC, MIC, and LIC  **SES indicator (level)**  PPP-aGDP), PPP-aTHE, unemployment rate (population) | First-ever incidence of stroke (assessment method not reported) | Lower PPP-aGDP correlated with higher incident risk of stroke (*ρ*= -0.661, p = 0.027; *R^2^* = 0.32, age-standardized for WHO World population; *ρ*= -0.553, p = 0.050; *R^2^* = 0.33, age-standardized for European population).  Lower PPP-aTHE correlated with higher incident risk of stroke (*ρ*= -0.623, p = 0.040; *R^2^* = 0.26, age-standardized for WHO World population; *ρ*= -0.587, p = 0.035; *R^2^* = 0.29, age-standardized for European population).  There were no correlations between unemployment rate and risk of stroke incidence (*ρ*= -0.492, p = 0.12, *R^2^* = 0.31, age-standardized for WHO World population; *ρ*= 0.102, p = 0.74, *R^2^* = 0.07, age-standardized for European population). | Unclear (Unclear if dual full-text review, no quality assessment of included studies) | | |
| *Cancers* | | | | |  |  |
| Adam et al., 2008 [[11](#_ENREF_11)] | **Population**  Children in HIC  **SES indicator (level)**  Education, occupation, income or employment (parental or household); housing characteristics; neighbourhood-level indicators | Incidence of childhood leukaemia (cancer registry, medical records) | Two studies showed an increased risk of leukaemia in children from deprived areas, 4 studies showed a decreased risk of leukaemia in children from deprived areas or lower SES, 1 study found SES not to be a determinant of leukaemia in children. | High (no extensive and systematic searches, unclear if dual study selection, no quality assessment of included studies) | | |
| Sidorchuk et al., 2009 [[26](#_ENREF_26)] | **Population**  General population in HIC and MIC  **SES indicator (level)**  Education, occupation, income or combined (individual, neighbourhood, population) | Incidence of lung cancer (assessment method not reported) | Risk for lung cancer incidence:  *Meta-analysis adjusted for smoking (low vs high)*  Education: RR 1.61, 95%CI: 1.40-1.85  Occupation: RR 1.48, 95%CI: 1.34-.1.65  Income: RR 1.37, 95%CI: 1.06-1.77  *HIC (low vs high)*  Education: RR 1.66, 95%CI: 1.10-2.51  Occupation: RR 1.42, 95%CI: 1.26-1.62  Income: RR 1.39. 95%CI: 1.13-1.69  Combined: RR 1.64, 95% CI: 0.83-3.25  *MIC (low vs high)*  Education: RR 1.66, 95%CI: 1.28-2.16  Occupation: RR 0.90, 95%CI: 0.66-1.23  Income: RR 1.30, 95%CI: 0.23-7.31  Combined: 0.59, 95%CI: 0.37-0.94 | Unclear (No in-depth or dual quality assessment of included studies) | | |
| Slatore et al., 2010 [[25](#_ENREF_25)] | **Population**  General population in the US  **SES indicator (level)**  Insurance status (individual) | Incidence of lung cancer (assessment method not reported) | 1 study found higher incidence rates of lung cancer for women and men from all age groups with Medicaid insurance (1.21 to 8.41) compared to non-Medicaid (0.27 to 4.34).  1 study found higher incidence rates of lung cancer for Medicare patients alone compared to Medicaid/  Medicare patients (IRR 1.77, 95%CI 1.52-2-06 to 2.00, 95%CI 1.49-2.69), but the effect was removed when the comparison group was restricted to patients covered by Medicaid >12 months before diagnosis. | Unclear (No dual quality assessment of included studies) | |  |
| Uthman et al., 2013 [[27](#_ENREF_27)] | **Population**  General population in HIC, MIC, and LIC  **SES indicator (level)**  Education, occupation, income or combined (individual) | Incidence of gastric cancer  (assessment method not reported) | Relative index of inequality² for gastric cancer incidence:  *Overall (low vs high)*  Education: RII 2.97, 95%CI: 1.92-4.58  Occupation: RII 4.33, 95%CI: 2.57-7.29  Income: RII 1.25, 95%: 0.93-1.68  Combined SEP: RII 2.64, 95%CI: 1.05-6.63  *HIC (low vs high)*  Education: RII 2.65, 95%CI 1.64-4.30  Occupation: RII 6.79, 95%CI 3.42-13.50  Income: RII 1.09, 95%CI: 0.76-1.56  Combined SEP: RII 4.50, 95%CI: 0.84-24.16  *MIC (low vs high)*  Education: RII 5.11, 95%CI 2.71-9.65  Occupation: RII 3.06, 95%CI 2.10-4.8  Income: RII 1.48, 95%CI: 0.61-3.58  Combined SEP: RII 1.36, 95%CI: 0.52-3.60 | Unclear (No quality assessment of included studies) | | |
| *Type 2 diabetes* | | | | |  |  |
| Agardh et al., 2011 [[28](#_ENREF_28)] | **Population**  General population in HIC, MIC, and LIC  **SES indicator (level)**  Education, occupation, or income (individual or household); country income [according to the World Bank’s country classification] (population) | Incidence of type 2 diabetes (self-reported, self-reported verified or diagnosed) | RR for type 2 diabetes incidence:  *Overall (low vs high)*  Education: RR 1.41, 95%CI: 1.28-1.51  Occupation: RR 1.31, 95%CI: 1.09-1.57  Income: RR 1.40, 95%CI: 1.04-1.88  *HIC (low vs high)*  Education: RR 1.45, 95%CI: 1.28-1.63  Occupation: RR 1.31, 95%CI: 1.05-1.63  Income: RR 1.40, 95%CI: 0.81-2.42  *MIC (low vs high)*  Education: RR 1.59, 95%CI: 1.28-1.97  Occupation: RR 1.27, 95%CI: 0.96-1.68  Income: RR 1.39, 95%CI: 1.06-1.82  *LIC (low vs high; n = 1)*  Education: –  Occupation: –  Income: RR 1.27, 95%CI: 0.99-1.62 | Unclear (Unclear if dual abstract review, no quality assessment of included studies) | | |
| Tamayo et al., 2010 [[18](#_ENREF_18)] | **Population**  General population in HIC and MIC  **SES indicator (level)**  Education, occupation (parental); childhood adversity^3^ | Incidence of type 2 diabetes in later life (self-reported, diagnosed) | 4 out of 6 studies showed a higher risk of type 2 diabetes in either girls or boys from low parental occupational status (adjusted point estimates of OR or HR for low vs. high SES ranging from 1.1, 95%CI 1.0-1.8 to-1.7, 95%CI 1.2-2.4 [w or m] or for most advantaged vs least advantaged OR 0.2, 95%CI 0.05-0.8 [m]), 2 studies showed no association (adjusted point estimates of OR for low vs. high SES 0.83, 95%CI, 0.5-1.5 to RR 1.08, 95%CI 0.95-1.2).  For education, 2 out of 3 studies showed a higher risk of type 2 diabetes in children from low SES (β 4.5, SE 0.78 and adjusted point OR for mothers beyond elementary level vs rest 0.6, 95%CI 0.5-0.8), and 1 showed no association (β 0.2, SE 0.1 [m] and 0.05, SE 0.1 [w]).  1 study showed no statistically significant association between type 2 diabetes incidence and childhood adversity (OR for mother: little interest in education 1.4, 95%CI 0.99-1.9). | High (Only one database searched, no dual quality assessment of included studies) | | |

| *Chronic respiratory diseases* |
| --- |

| Gershon et al., 2012 [[29](#_ENREF_29)] | Population  General population in HIC  SES indicator (level)  Education, occupation, and/or income (na); Townsend Deprivation Score^1^ (neighbourhood) | Prevalence and incidence of COPD (assessment method not reported) | Majority of studies (6 out of 8) found individuals of the lowest SES strata more likely to have or develop COPD than those of the highest (point estimates of OR ranging from 0.8 – 3.7, RII ranging from 2.2 to 3.2). | Low |
| --- | --- | --- | --- | --- |
| RR = Relative risk; CI = Confidence interval; SES = Socioeconomic status; COPD = Chronic obstructive pulmonary disease; OR = Odds ratio; RII = Relative index of inequality; na = not  available; SEP = Socioeconomic position; y = years; vs = versus; p = p-value; LMIC = Low and middle income countries; HIC = High income countries; MIC = Middle income countries; LIC = Low income countries; PAD = Peripheral artery disease; MI = Myocardial infarction; CVD = Cardiovascular diseases; CHD = Coronary heart disease; IHD = Ischaemic heart disease; IMT = Intima-media thickness; AP = Angina pectoris; PPP-aGDP = Per capita GDP adjusted for purchasing power parity; PPP-aTHE = total health expenditures per capita at purchasing power Parity; *ρ* = Spearman rank correlation coefficient; *R²* = Effect size; IRR = Incidence rate ratio; HR = Hazard ratio; WHO = World Health Organization; n = number of studies; w = women; m = men; β = Regression coefficient; NCDs = Non-communicable diseases;  ^1^Townsend Deprivation Score is a measure derived from unemployment, car ownership, home ownership and overcrowding  ^2^Relative Index of Inequality is an indicator of the degree of inequality across socioeconomic categories  ^3^Questionnaire including abuse, physical and emotional neglect, household dysfunction | | | | |
