# Supplementary material for: Socioeconomic inequalities in non-communicable diseases and their risk factors: an overview of systematic reviews
Source: BMC Public Health. 2015 Sep 18;15:914. doi: 10.1186/s12889-015-2227-y (PMC4575459; doi:10.1186/s12889-015-2227-y)
Supplement: Additional file 3: — Study characteristics and summary of results for adverse outcomes from NCDs. (DOCX 39 kb) [file 12889_2015_2227_MOESM3_ESM.docx]

Additional file 3: Study characteristics and summary of results for adverse outcomes from NCDs

| Author, year | | Search Databases  Population  SES indicator (level) | | | Outcome | Results | Risk of Bias | |
| --- | --- | --- | --- | --- | --- | --- | --- | --- |
| *Cardiovascular diseases* | | | | | | | |  |
| Calvillo-King et al., 2012 [[30](#_ENREF_30)] | | **Population**  Patients with heart failure in HIC  **SES indicator (level)**  Education, SES, insurance (na), distance to hospital (neighbourhood) | | | Mortality in heart failure after hospitalisation (30 day) | Risk of mortality in heart failure:  Lower vs higher education: RR 1.05, 95%CI: 0.98-1.12 (n = 1)  Lower vs higher neighbourhood SES: RR 1.13, 95%CI: 0.92-1.38 (n = 1)  Medicaid insurance vs other: OR 0.66, 95%CI: 0.3-1.4 (4 studies, result from one)  ≤ 25 miles to hospital vs > 25 miles to hospital: OR 0.95, 95%CI: 0.92-0.98 (n = 1) | Unclear (No quality assessment of included studies) | |
| Feigin et al., 2009 [[24](#_ENREF_24)] | | **Population**  Patients with stroke in HIC and LMIC  **SES indicator (level)**  Country income [according to the World Bank’s country classification] (population) | | | Early case fatality of stroke (21 day to 1 month) | Early case fatalities of total strokes (%):  *HIC*  - 1970-79: 35.9%  - 1980-89: 21.5%  - 1990-99: 22.2%  - 2000-08: 19.8%  →non-significant reduction  *LMIC*  - 1980-89: 35.2%  - 1990-99: 23.0%  - 2000-08: 26.6%  →non-significant reduction | Unclear (No quality assessment of included studies) | |
| Galobardes et al., 2006 [[15](#_ENREF_15)] | | **Population**  General population mainly from HIC  **SES indicator (level)**  Education, occupation, wealth, employment, social class (maternal, paternal, parental), housing conditions, overcrowding, number of siblings, farm size, car ownership(household); maternal marital status, single-parent family and other indirect SES measures | | | Overall CVD, CHD (MI, IHD), stroke, angina, other CVD subtypes (PAD, markers of atherosclerosis, rheumatic heart disease) mortality | 19 out of 24 prospective studies found an association between low childhood SES and increased risk CVD mortality. In 5 out of 9 studies the association was stronger for stroke than CHD. | High (Search databases not mentioned, no information on review process, no quality assessment of included studies) | |
| Galobardes et al., 2004 [[10](#_ENREF_10)] | | **Population**  General population mostly in HIC  **SES indicator (level)**  Education (parental), occupation (paternal), housing conditions, overcrowding, number of siblings, home ownership, farm size (household); maternal marital status and presence of both natural parents during childhood | | | Overall CVD, CHD (MI, IHD), stroke, overall cancer, lung cancer, other cancers, COPD mortality | 5 out of 9 studies found a higher risk of overall CVD mortality among those with low childhood SES, with results generally remaining statistically significant after adjustment for adult SES and/or adult CVD risk factors.  7 out of 10 studies found a higher risk of CHD mortality among those with low childhood SES, although adult SES attenuated the association in some studies.  4 out of 6 studies found a higher risk of stroke mortality among those with low childhood SES.  4 out of 5 studies found no association between overall cancer mortality and childhood SES, and the effect was removed by adjustment for adult SES in the remaining study.  3 out of 3 studies found a higher risk of lung cancer mortality among those with low childhood SES, although the association was largely explained by adults SES in 2 studies. 1 study showed no association of childhood SES with a group of other smoking-related cancers.  1 study found a higher risk of stomach cancer mortality among those with low childhood SES, independent of adult circumstances.  1 study found a higher risk of large-bowl and rectal cancer among those who had the poorest housing conditions during childhood.  There was no association between non-smoking related cancers (3 studies), prostate cancer (1 study) and malignant melanoma (1 study) mortality and childhood SES.  1 study did not find an association between higher COPD mortality and overcrowding. | High (Search databases not mentioned, no information on review process, no quality assessment of included studies) | |
| Pollitt et al., 2005 [[17](#_ENREF_17)] | | | **Population**  General population and patients with CVD and stroke from HIC  **SES indicator (level)**  Education, occupation, employment (parental/paternal); housing conditions, family size, perceived wealth, family situation, farm size (household) and other indirect SES measures | CVD (MI, IHD, carotid IMT; CHD, AP), stroke mortality | | 11 out of 13 studies found a higher risk of CVD mortality among those with low childhood SES. Most associations remained statistically significant after adjustment for CVD risk factors and/or adult SES.  3 out of 3 studies showed a higher risk of stroke mortality among those with low childhood SES. Adjustment for adjustment for CVD risk factors and/or adult SES had minor impact on the effect.  5 out of 5 studies reported an association between cumulative life course exposure to low SES conditions and increased CVD mortality. | High (Only one database searched) | |
| Sposato et al., 2012 [[21](#_ENREF_21)] | **Population**  Patients with stroke in HIC, MIC, and LIC  **SES indicator (level)**  PPP-aGDP, PPP-aTHE, unemployment rate (population) | | | 30-day case-fatality rates of stroke; intracerebral hemorrhages | | Lower PPP-aGDP correlated with higher 30-day case-fatality rates of stroke (*ρ* = -0.713, p < 0.001; *R^2^* = 0.43) and a greater proportion of intracerebral hemorrhages (*ρ* = -0.689, p < 0.001; *R^2^* = 0.43).  Lower PPP-aTHE correlated with higher 30-day case-fatality rates of stroke (*ρ* = -0.701, p < 0.001; *R^2^* = 0.45) and a greater proportion of intracerebral hemorrhages (*ρ* = -0.643, p < 0.001; *R^2^* = 0.32).  There was no correlation between unemployment and 30-day case-fatality rates of stroke (*ρ*= 0.204; p = 0.32, *R^2^* = 0.04) and proportion of intracerebral hemorrhages (*ρ* = -0.258, p = 0.18; *R^2^* = 0.04). | Unclear (Unclear if dual full-text review, no quality assessment of included studies) | |
| *Cancers* | | | | | | | |  |
| Canturk et al., 2010 [[31](#_ENREF_31)] | | **Population**  Patients with retinoblastoma in upper middle, lower middle and low income countries  **SES indicator (level)**  Country income [according to the World Bank’s country classification] (population) | | | Survival of retinoblastoma | Estimated survival of retinoblastoma:  *Upper middle income countries*  79% (range, 54-93%)  *Lower middle income countries*  77% (range, 60-92%)  *Low income countries*  40% (range, 23-70%)  p = 0.001 | Unclear (Unclear if dual full-text review, no quality assessment of included studies) | |
| Galobardes et al., 2004 [[10](#_ENREF_10)] | | **Population**  General population mostly in HIC  **SES indicator (level)**  Education (parental), occupation (paternal), housing conditions, overcrowding, number of siblings, home ownership, farm size (household); maternal marital status and presence of both natural parents during childhood | | | Overall cancer, lung cancer, and other cancers mortality | 4 out of 5 studies found no association between overall cancer mortality and childhood SES, and the effect was removed by adjustment for adult SES in the remaining study.  3 out of 3 studies found a higher risk of lung cancer mortality among those with low childhood SES, although the association was largely explained by adults SES in 2 studies. 1 study showed no association of childhood SES with a group of other smoking-related cancers.  1 study found a higher risk of stomach cancer mortality among those with low childhood SES, independent of adult circumstances.  1 study found a higher risk of large-bowl and rectal cancer among those who had the poorest housing conditions during childhood.  There was no association between non-smoking related cancers (3 studies), prostate cancer (1 study) and malignant melanoma (1 study) mortality and childhood SES. | High (Search databases not mentioned, no information on review process, no quality assessment of included studies) | |
| Gorey et al., 2009 [[20](#_ENREF_20)] | | **Population**  Patients with breast cancer in the US and Canada  **SES indicator (level)**  Income (neighbourhood) | | | Breast cancer survival | Within Canada, there was no association between area-SES and breast cancer survival, a little survival disadvantage was only observed for lowest income areas compared to highest income areas (pooled RR 0.94, 95%CI 0.93-0.95).  Within the US, breast cancer survival was consistently associated with area-SES. Women with breast cancer from low and middle income areas had survival disadvantage compared to women from high income areas (pooled RR ranging from 0.73, 95%CI 0.72-0.74 for low to 0.96, 95%CI 0.94-0.98 for middle income area compared with high income areas). | High (Only one database searched, no information on review process, no quality assessment of included studies) | |
| Slatore et al., 2010 [[25](#_ENREF_25)] | | **Population**  Patients with lung cancer in the US  **SES indicator (level)**  Insurance status (individual) | | | Lung cancer mortality | 4 out of 4 studies showed a higher risk for lung cancer mortality for Medicaid insurance compared to other or private insurance.  2 studies showed mixed results on the association between Medicare vs Medicaid/Medicare and lung cancer mortality.  1 study showed a higher risk for lung cancer mortality for Medicare insurance and no insurance compared to private insurance.  2 studies showed no association between lung cancer mortality and insurance status (other vs private and commercial vs other).  1 study found mixed results for lung cancer mortality and different Medicare schemes. | Unclear (No dual quality assessment of included) | |
| *Chronic respiratory diseases* | | | | | | | |  |
| Galobardes et al., 2004 [[10](#_ENREF_10)] | | **Population**  General population mostly in HIC  **SES indicator (level)**  Education, occupation, wealth (parental) , housing conditions, overcrowding, number of siblings, residence in orphanage (household); maternal marital status or illegitimacy and other indirect SES measures | | | COPD mortality | 1 study did not find an association between higher COPD mortality and overcrowding in childhood. | High (Search databases not mentioned, no information on review process, no quality assessment of included studies) | |
| Gershon et al., 2012 [[29](#_ENREF_29)] | | **Population**  Patients with COPD in HIC  **SES indicator (level)**  Education, occupation and/ or income (na) | | | COPD mortality | Individuals of the lowest SES consistently had significantly higher mortality from COPD than those of the highest (point estimates of rate ratio ranging from 1.1-10.8, RR ranging from 1.9-2.5), except for 1 study (out of 5) where income was not associated with COPD mortality (OR 0.8, 95%CI 0.5-1.3). | Low | |
| SES = Socioeconomic status; COPD = Chronic obstructive pulmonary disease; na = not available; RR = Relative risk; OR = Odds ratio; CI = Confidence interval; p = p-value; n = number of studies; US = United States; PPP-aGDP = Per capita GDP adjusted for purchasing power parity; PPP-aTHE = total health expenditures per capita at purchasing power parity; *ρ* = Spearman rank correlation coefficient; *R²* = Effect size; y = years; NCDs = Non-communicable diseases; vs = versus; LMIC = Low and middle income countries; HIC = High income countries; MIC = Middle income countries; LIC = Low income countries; MI = Myocardial infarction; CVD = Cardiovascular diseases; CHD = Coronary heart disease; IHD = Ischaemic heart disease; IMT = Intima-media thickness; AP = Angina pectoris; | | | | | | | | |
