# Supplementary material for: Socioeconomic inequalities in non-communicable diseases and their risk factors: an overview of systematic reviews
Source: BMC Public Health. 2015 Sep 18;15:914. doi: 10.1186/s12889-015-2227-y (PMC4575459; doi:10.1186/s12889-015-2227-y)
Supplement: Additional file 4: — Study characteristics and summary of results for NCD risk factors. (DOCX 27 kb) [file 12889_2015_2227_MOESM4_ESM.docx]

Additional file 4: Study characteristics and summary of results for NCD risk factors

| Author, year | Search Databases  Population  SES indicator (level) | Outcome | Results | Risk of Bias |
| --- | --- | --- | --- | --- |

| *Obesity* |
| --- |

| Ekpenyong and Akpan, 2013 [[19](#_ENREF_19)] | Population  Adults in Nigeria  SES indicator (level)  SES (na) | Prevalence of overweight and obesity (assessment method not reported) | | Prevalence:  *Adults (overweight [obesity]) in urban area (n = 1)*  Low SES: 24.8% [12.9%]  Medium SES: 18.9% [5.65%]  High SES: 14.6% [4.86%] | High (No extensive searches, unclear if dual study selection, no quality assessment of included studies) |
| --- | --- | --- | --- | --- | --- |
| Papandreou et al., 2008 [[13](#_ENREF_13)] | **Population**  Children and adults in Mediterranean countries  **SES indicator (level)**  GNI per capita [according to the World Bank’s country classification] (population) | | Prevalence of obesity (BMI ≥30, assessment method not reported) | Prevalence(Median):  *Children*  HIC vs MIC: 11.50% vs 3.90% (p = 0.071) [m]; 7.20% vs 3.20% (p = 0.074) [w]  *Adults*  HIC vs MIC: 20.10% vs 22.00% (p = 0.62) [m]; 24.35% vs 30.15% (p = 0.368) [w] | High (Only one database search, no quality assessment of included studies) |
| Shrewsbury and Wardle, 2008 [[12](#_ENREF_12)] | **Population**  Children in HIC  **SES indicator (level)**  Education, occupation (parental), income (household), neighbourhood SES | | Prevalence of childhood obesity (BMI from measured or self-reported height and weight) | In bivariate analyses:  19 out of 45 studies found higher obesity prevalence rates among those with low SES (all indicators), 12 studies found no and 14 varied associations between all SES indicators and obesity  15 out of 20 studies found higher obesity prevalence rates among those with low education, 1 study found no, and 4 varied associations between education and obesity  5 out of 13 studies found higher obesity prevalence rates among those with low occupation, 6 studies found no, and 2 varied associations between occupation and obesity  4 out of 11 studies found higher obesity prevalence rates among those with low income, 3 studies found no, and 4 varied associations between income and obesity  2 out of 5 studies found higher obesity prevalence rates among those with low SES (composite measures), 1 study found no, and 2 varied associations between composite measures of SES and obesity  2 out of 7 studies found higher obesity prevalence rates among those with low neighbourhood SES, 3 studies found no, and 2 varied associations between neighbourhood SES and obesity | High (Only one database searched, unclear if dual abstract and full-text review, no quality assessment of included studies) |
| Tamayo et al., 2010 [[18](#_ENREF_18)] | **Population**  General population in HIC  **SES indicator (level)**  Education, occupation or income (parental) | | Prevalence or incidence of overweight and obesity in later childhood/life (BMI from measured or self-reported height and weight; fat mass measured by [DXA]) | 5 studies showed no direct or a small influence of education on later childhood overweight and obesity (adjusted point estimates of OR ranging from 0.96, 95%CI 0.7-1.4 to-1.4, 95%CI 0.8-2.4, β 0.007, SE: 0.003), 2 studies showed an increased risk for overweight and obesity in the lowest education strata (adjusted point estimates of SII^1^ 1.21, 95%CI 1.08-1.36 [m], 1.34, 95%CI 1.23-1.46 [w]; β 0.481, SE: 0.152).  No or small associations between occupation and overweight or obesity were found in 4 studies (adjusted point estimates of OR ranging from 1.04, 95%CI 0.9-1.2 to-1.10, 95%CI 1.07-1.1, β -0.05, SE: 0.08 to-0.48, SE: 0.17), of which 2 reported on adult overweight and obesity. 2 studies reported an increased risk of later childhood overweight and obesity in the lowest occupation strata (adjusted point estimates of OR 2.8, 95%CI 1.6-5.2 [w only], and 2.4, 95%CI 1.02-5.4)  3 studies observed effects regarding income discrepancies and later childhood overweight and obesity (adjusted point estimates of RR, OR in lowest income group OR 2.5, 95%CI 1.3-4.8 and RR 2.8, 95%CI 1.4-5.8, β -2.6, 95%CI -3.8 to -1.3), 2 studies showed no associations (adjusted point estimate of OR 1.0, 95%CI: 0.99-1.00, β 0.003, SE: 0.001) | High (Only one database searched, no dual quality assessment of included studies) |
| Wang and Beydoun, 2007 [[14](#_ENREF_14)] | **Population**  US adults and children  **SES indicator (level)**  Not reported for adult data; poverty income ratio for children data (household) | | Prevalence of overweight and obesity (BMI from measured height and weight) | Prevalence ratios (NHANES² data):  *Adults (obesity)*  Low vs high SES:  -1971-74: 1.6, 14.4% vs 7.4% (m); 3.4, 24.9% vs 7.3% (w)  -1999-2000: 1.1, 29.4% vs 23.6% (m); 1.3, 37.8% vs 29.9% (w)  *Children aged 2-9 y (overweight)*  Low vs high SES:  -1971-75: 1.9, 6.0% vs 3.2% (m); 0.8, 3.2% vs 4.2% (f)  -1999-2002: 1.8, 17.4% vs 9.7% (m); 1.0, 11.9% vs 11.4% (f)  *Children aged 10-17 y (overweight)*  Low vs high SES:  -1971-75: 0.8, 4.1% vs 5.1% (m); 2.0, 7.4% vs 3.7% (f)  -1999-2002: 1.1, 17.3% vs 15.9% (m), 1.6, 20.0% vs 12.9% (f) | High (Only one database searched, no information on review process, no quality assessment of included studies) |
| OR = Odds ratio; SII = Slope index of inequality; m = men or male; w = women; f = female; SES = Socioeconomic status; BMI = Body mass index; DXA = Dual-energy X-ray absorptiometry; na = not available; vs = versus; US = United States; CI = Confidence interval; SE = Standard error; RR = Relative risk; SII = Slope index of inequalities; y = years; n = number of studies; GNI = Gross national income; HIC = High income country; MIC = Middle income country; β = Regression coefficient;  ^1^Slope Index of Inequalities (method comparable to linear regression)  ²National Health and Nutrition Examination Surveys (USA) | | | | | |
